# The assessment of the kinematics of the rescuer in continuous chest compression during a 10-min simulation of cardiopulmonary resuscitation

**DOI:** 10.1186/s40001-019-0369-6

**Published:** 2019-02-08

**Authors:** Bogusław Bucki, Dariusz Waniczek, Robert Michnik, Jacek Karpe, Andrzej Bieniek, Arkadiusz Niczyporuk, Joanna Makarska, Tomasz Stepien, Dariusz Myrcik, Hanna Misiołek

**Affiliations:** 1 Wydział Zdrowia Publicznego w Bytomiu, Śląski Uniwersytet Medyczny w Katowicach, Katowice, Poland; 2 Department of Propaedeutics Surgery, Chair of General, Colorectal and Polytrauma Surgery, Medical University of Silesia in Katowice, Bytom, ul. Żeromskiego 7, 41-902 Katowice, Poland; 3 Wydział Inżynierii Biomedycznej w Zabrzu, Politechnika Śląska w Gliwicach, Katowice, Poland; 4 Wydział Lekarski z Oddziałem Lekarsko–Dentystycznym w Zabrzu, Śląski Uniwersytet Medyczny w Katowicach, Katowice, Poland

**Keywords:** Kinematics, Cardiopulmonary resuscitation, Cardiac arrest

## Abstract

**Background:**

In pursuit of improvement in cardiopulmonary resuscitation (CPR), new technologies for the measurement and assessment of CPR quality are implemented. In our study, we assessed the kinematics of the rescuer during continuous chest compression (CCC–CPR). The proper performance of the procedure is a survival predictor for patients with cardiac arrest (CA). The purpose of the study was a prospective assessment of the kinematics of the rescuer’s body with consideration given to the depth and rate of chest compression (CC) as the indicator of properly performed CC maneuver by professional and non-professional rescuers during a simulation of a 10-min CCC using a manikin.

**Methods:**

Forty participants were enrolled in the study. CCC–CPR was performed in accordance with the 2015 AHA guidelines on a manikin positioned on the floor. Kinematic data on the movement were obtained from the measuring system (X-sens MVN Biomech) transmitting information from 17 inertial sensors. Measurement data were imported to the author’s program RKO-Kinemat written in the Matlab and C # environments. Two groups of results were distinguished: Group I—results of CC with the depth of ≥ 40 mm and Group 2—CC results with the depth of < 40 mm.

**Results:**

The multiple regression model demonstrated that the path length, left knee flexion angle, and left elbow flexion angle were the essential elements of the rescuer’s kinematics that facilitated achieving and maintaining the normal depth of CC.

**Conclusions:**

We believe that raising the rescuer’s hips by moving the center of the rescuer’s body over the point of sternal compression increases the value of the CC force vector, thereby increasing the depth of CC. In addition, we observed that, during an effective CC, the rescuer was unable to maintain arms straight and, in consequence, a slight elbow flexion was observed. It, however, did not influence the quality of the maneuver.

## Background

Cardiopulmonary resuscitation (CPR) is a recognized method of basic life support (BLS) in persons with sudden cardiac arrest (CA). The guidelines of the 2015 American Heart Association (AHA) and the European Resuscitation Council (ERC) emphasize the importance of the early initiation of BLS [1–5]. Attention is also paid to the fact that, within the first minutes after CA, even non-professional rescue procedures, particularly effective chest compression (CC), may determine the survival of patients with CA. It is also suggested that, to encourage a greater number of individuals to initiate the early resuscitation, only CC should be performed [1, 2, 4–12]. The effectiveness of this procedure is comparable to the standard CPR due to similar survival rates and a decreased number of post-resuscitation neurological deficits in persons with CA [2, 3, 5–7, 13–18]. Therefore, the alternative procedure which is continuous CC CPR (CCC–CPR) done within the first 10 min of resuscitation may be a predictor of patient survival. A number of researchers indicate the particular importance of the quality of the CC maneuver, especially the depth of sternal compression of at least 50 mm or the importance of a fully recoiled chest after each compression, maintenance of the recommended rate of compression of at least 100–120/min, and minimization of the interruptions for rescue breaths or defibrillation [1, 2, 4, 9, 15–20]. The concept is reflected in a change in the sequence of the maneuver during CPR from ABC (airway, breath, and circulation) to CAB (circulation, airway, and breath) [5, 6, 16].

**Fig. 1:**
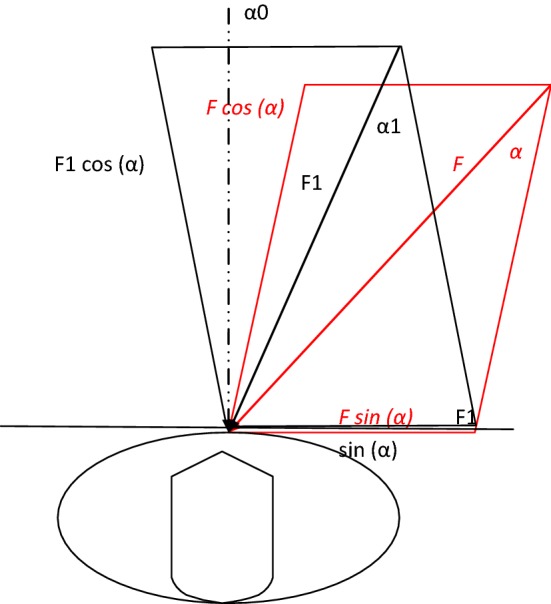
Model distribution of forces in chest compression. *F* force of chest compression applied at the α angle, *Fcos(α)* value of the vertical force for *α* = 105°, *F1* force of chest compression applied at the *α*1 angle, *Fcos(α1)* value of the vertical force for *α*1 = 97°

From the biomechanical perspective, obtaining the normal CC requires overcoming resistance forces resulting from chest rigidity [21]. Consequently, the direction of compression force is significant for proper CC. To obtain the optimal CC, the compression should be similar to the sagittal axis and the energy transferred on the sternum should result in the most perpendicular compression of the sternum (Table 1, Fig. 1).

**Table 1:** Model changes in the values of the compression force, depending on the angle of the force vector deviation

*α*	cos(*α*)	*F*
0°	1	1 × *F*
5°	0.9962	0.9962 × *F*
10°	0.9848	0.9848 × *F*
15°	0.9659	0.9659 × *F*
20°	0.9397	0.9397 × *F*
25°	0.9063	0.9063 × *F*

*α* angle of application of force, *F* chest compression force

Such a procedure allows the transfer of compression energy on a selected point on the chest, thereby possibly reducing the likelihood of accidental fractures of chest bone structures. It prevents severe thoracic or abdominal trauma [19, 22]. To improve the quality of CPR, it is necessary to discover and implement new technologies for the measurement and assessment of CPR quality and new control devices integrated with the training equipment, which allows receiving real-time feedback at the time of the maneuver [9, 22–24].

Searching for factors which influence the correct performance of CPR, we planned the experiment that would indicate the elements of the rescuer’s body movement affecting the quality of the performed CC at the time of the maneuver.

The aim of the study was a prospective assessment of the kinematics of the rescuer, with consideration given to the depth of CC as the indicator of the performance of CC maneuver done by professional and non-professional rescuers during a simulation of a 10-min CCC using a manikin.

## Methods

**Fig. 2:**
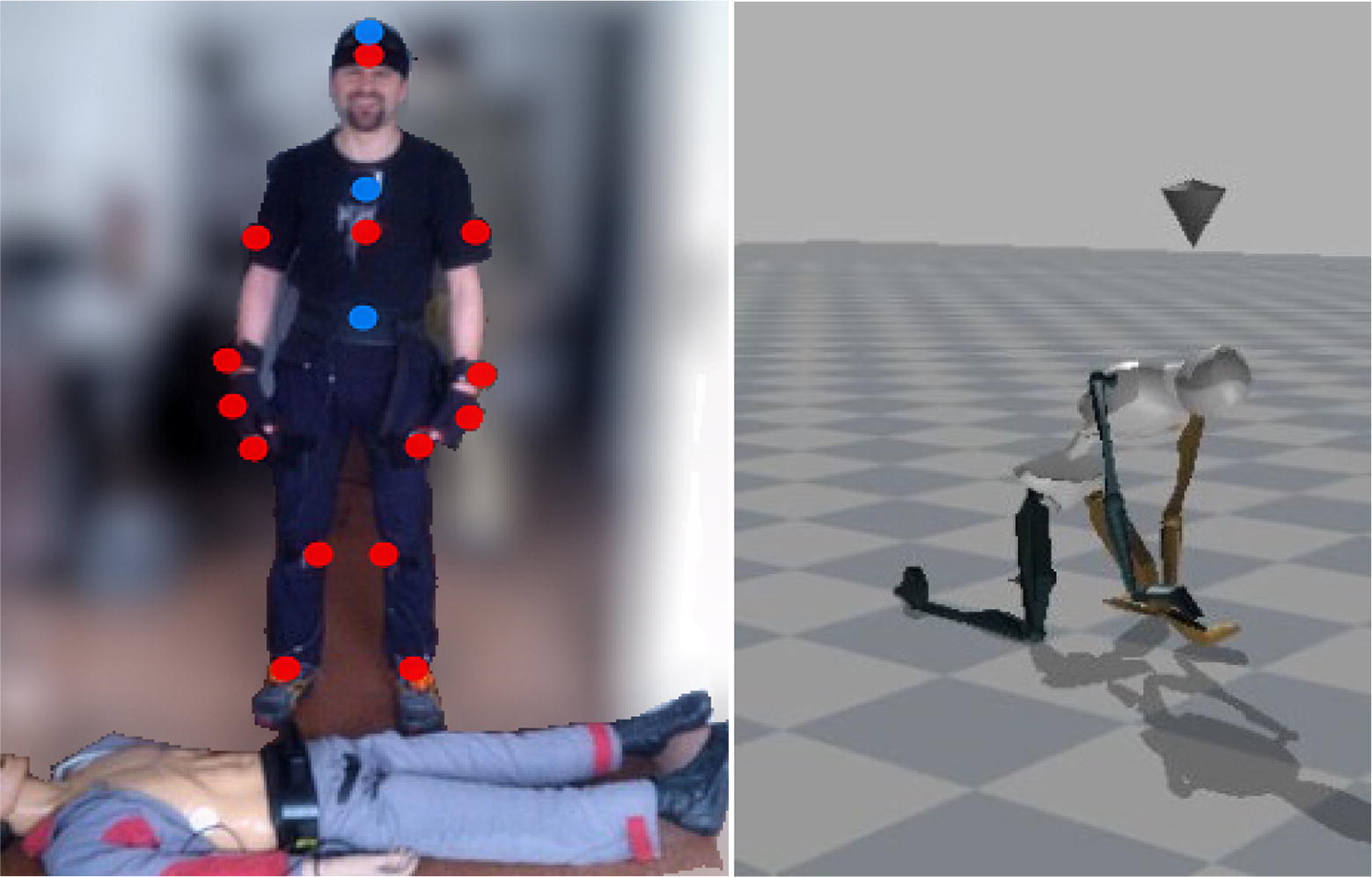
The study participant wearing the Xsens MVN Biomech suit and Avatar—electronic imaging of the rescuer’s posture based on signals obtained from inertial sensors of the suit. Big, red point—front of the rescuer; big blue point—back of the rescuer (invisible)

Forty volunteers were enrolled in the study, i.e., 10 professional medical rescuers, 22 medical rescue students, and 8 rescue physicians. The participation in the study was voluntary. During the simulation, each participant performed CCC–CPR in accordance with the 2015 AHA guidelines. The simulation was performed in a closed room with a constant temperature. The participants did not receive feedback on the time elapsed or the correctness of the maneuver. The manikin was positioned on the floor and each rescuer performed CC in a kneeling position on the right side of the manikin. The participants were instructed on the necessity of performing CCC–CPR until the end-point was reached (time lapse of 15 min or refusal to continue the trial). The simulation was performed on the manikin (Ambu Cardiac Care Man) with the compression resistance of 9.0 N/cm^2^. Data on the continuous recording of the depth and rate of CC were transferred in real time to the computer memory via the interface. Kinematic data on the movement of the rescuer during CCC–CPR were obtained from the measuring system (X-sens MVN Biomech), which transfers information from 17 inertial sensors placed on the body of the rescuer performing CCC–CPR (Fig. 2). The sensors were placed on the head, in the middle of the chest, between the shoulder blades, on hands, forearms, arms, feet, and lower and upper legs.

The planned sensor placement facilitated the determination of the position of the forearm, arm, upper leg, lower leg, and trunk. The measurement system also allowed to determine flexion angles of the limbs and spinal segments, and the analysis of the deviation of C7-Th1 spinal segments in sagittal and horizontal planes from the sagittal axis which determines the path length (PL) and the ellipse area (EA) based on the total length of the indicated segments and the area indicated by the sensor C7-Th1. The results of the kinematic measurements were recorded at the beginning of each minute, for 30 s. The obtained measurement data were imported into the author’s program (RKO-Kinemat) written in the MATLAB and C# environments. The program facilitated synchronization and visualization of the data from both measurement systems. The measurement data on the depth and rate of CC were analyzed by determining the mean values recorded at 60-s intervals. The angles were defined in accordance with the guidelines of the International Society of Biomechanics.

The analysis of the results was performed based on the complete data obtained in real time within the period of 10 min during which the trial was performed. On the basis of the study results, two groups of results were distinguished, i.e., Group 1—CC results with the depth ≥ 40 mm and Group 2—CC results with the depth < 40 mm.

For the assessment of the axis of the CC force vector, we applied the principle modeled on the analysis of the movement of feet center of pressure (COP) on the support plane determining PL and EA, which includes 95% of COP positions based on the assessment of the rescuer’s movement of the C7-Th1 segment during CCC–CPR. In our study, the direction of the CC force vector was determined by the line designated by the signals from accelerometers placed in the middle of the right palm placed over the dominant hand, i.e. left hand (placed directly on the manikin chest) and above the C7-Th1 segment of the rescuer’s spine. As the force application point (i.e., the center of the palm) should be considered constant, PL and EA indicated during CCC–CPR allowed to assess the deviation of the CC force vector from the sagittal axis and the extent of the movement of the shoulder girdle (expressed by the movement of C7-Th1 spinal segment) in the planes parallel to horizontal and sagittal planes of the manikin’s chest. The assessment of the performance of the maneuver was done based on the comparison of CC depths measured at 60-s intervals. The correlation of the results with the kinematic measurements allowed to determine to what extent the range of changes of the studied biomechanical parameters affected the expected quality of CCC–CPR during rescue procedures. The analysis of the kinematic parameters of the rescuer’s movement during CC was based on the criterion of achieving at least 40-mm depth of sternal compression during the CCC–CPR simulation.

### Statistical methods

Statistical analysis was performed based on data analysis software system STATISTICA, v.10, StatSoft, Inc. (2011). After estimating the sample distribution with the Shapiro–Wilk test, multivariate analysis of variance (MANOVA) was applied for repeated measurements with the post hoc Bonferroni test to compare time results in the examined groups. In the analysis of differences between the examined variables, Student’s *t* test was applied for variables of normal distribution and the Mann–Whitney *U* test was applied for variables with distribution other than normal. The data were presented as the mean and the standard error. The values of *p* < 0.05 were considered statistically significant. Multiple regression analysis was used to assess the influence of individual kinematic variables on the depth of the CC performed by the rescuers.

## Results

Table 2 presents the multiple regression analysis of the influence of kinematic factors on the CC depth. The examined regression model explaining the CC depth proved to be relevant (*F* = 58.200; *p* < 0.001) and the selected predictors totally accounted for 41.8% of the dependent variable (*R*^2^ = 0.418). Four of seven predictors included in the model had a significant influence on the CC depth, i.e., time (T)—(*β* = 0.36, *t* = 8.20, *p* < 0.001); PL—(*β* = 0.29, *t* = − 6.53, *p* < 0.001), left elbow flection angle (LEFA)—(*β* = 0.24, *t* = − 5.25, *p* < 0.001); left knee flexion angle (LKFA)—(*β* = 0.29, *t* = 6.23, *p* < 0.001) (Table 2).

**Table 2:** Multiple regression analysis of the influence of the analyzed factors on the depth of chest compression

	*β*	Standard error of *β*	*β*	Standard error of *β*	*t*(314)	*p*
Intercept			− 67.7183	3.438446	− 19.6945	0.000
Rate	0.363932	0.044381	0.1459	0.017791	8.2003	0.000
PL	− 0.293873	0.044983	− 0.0017	0.000254	− 6.5330	0.000
LKFA	0.290902	0.046699	0.1638	0.026298	6.2293	0.000
LEFA	− 0.239073	0.045544	− 0.1598	0.030445	− 5.2492	0.000

Corrected *R*^2^ − 0.418; *F*(4.13) − 58.200; *p* = 0.000; standard error of estimation = 4.712

*PL* path length, *LKFA* left knee flexion angle, *LEFA* left elbow flection angle

When all the relevant factors were considered, the regression equation was as follows:

$$ Y\, = \, - \,67\, + \,0.36\, \times \,\left( C \right)\, - \,0.29\, \times \,\left( {\text{PL}} \right) - \,0.24\, \times \,\left( {\text{LEFA}} \right)\, + \,0.29\, \times \,\left( {\text{LKFA}} \right). $$

**Fig. 3:**
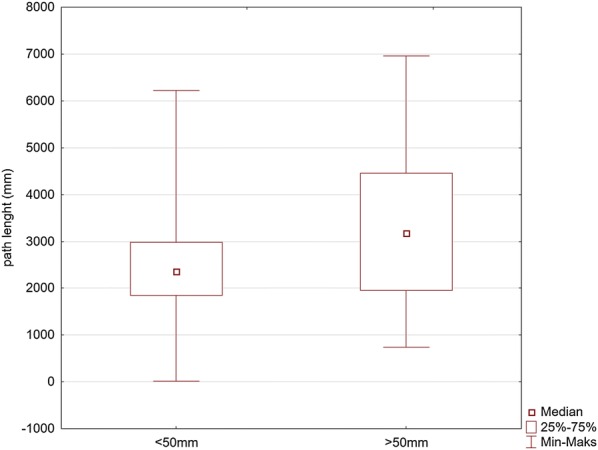
Comparison of the values of the path length for the maneuver with deep (> 50 mm) and too shallow (< 50 and) chest compression

The PL median for the CC depth > 40 mm was 2971 mm (min = 203, max = 6954) and was significantly higher compared to 2282 mm (min = 18, max = 4864) in the remaining group of the results (*p* = 0.000) (Fig. 3).

**Fig. 4:**
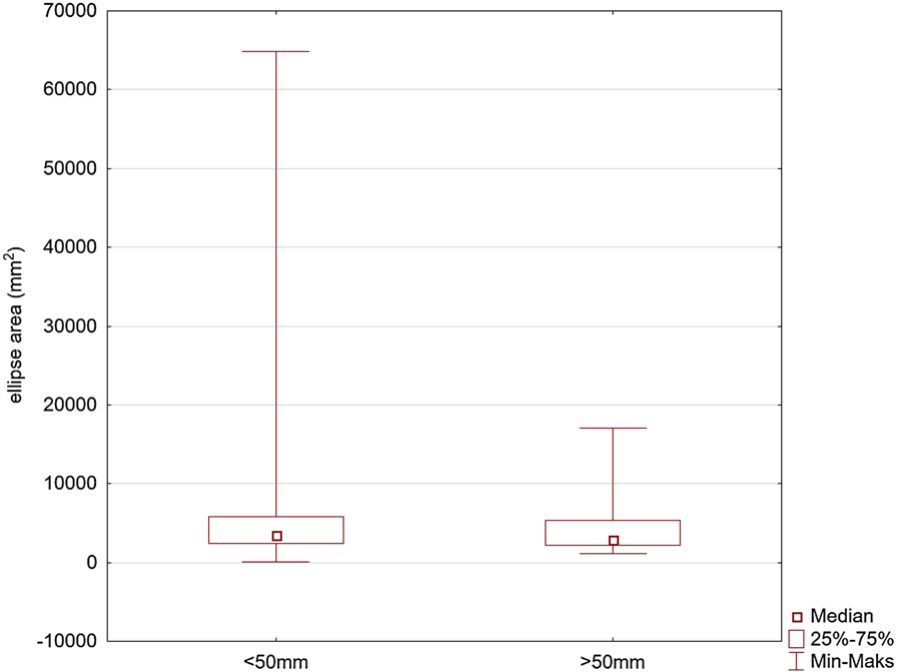
Comparison of the values of the ellipse area for the maneuver with deep (> 50 mm) and too shallow (< 50 mm) chest compression

The EA median for the CC depth > 40 mm was 3732 mm (min = 1107, max = 64,864) and was not significantly higher compared to 3302 mm (min = 74, max = 38,575) in the remaining group of the results (*p* = 0.186) (Fig. 4).

**Fig. 5:**
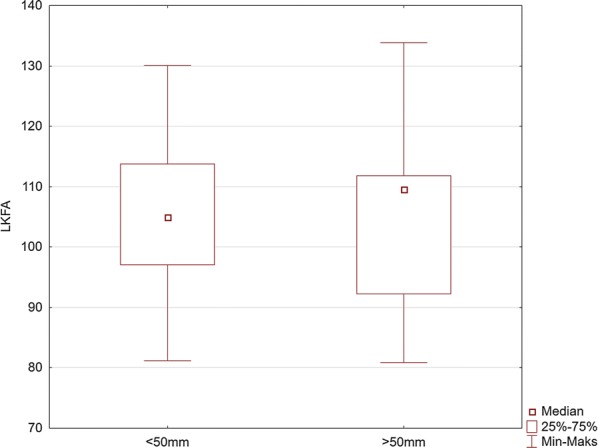
Comparison of the values of the left knee flexion angle (LKFA) for the maneuver with deep (> 50 mm) and too shallow (< 50 mm) chest compression

**Fig. 6:**
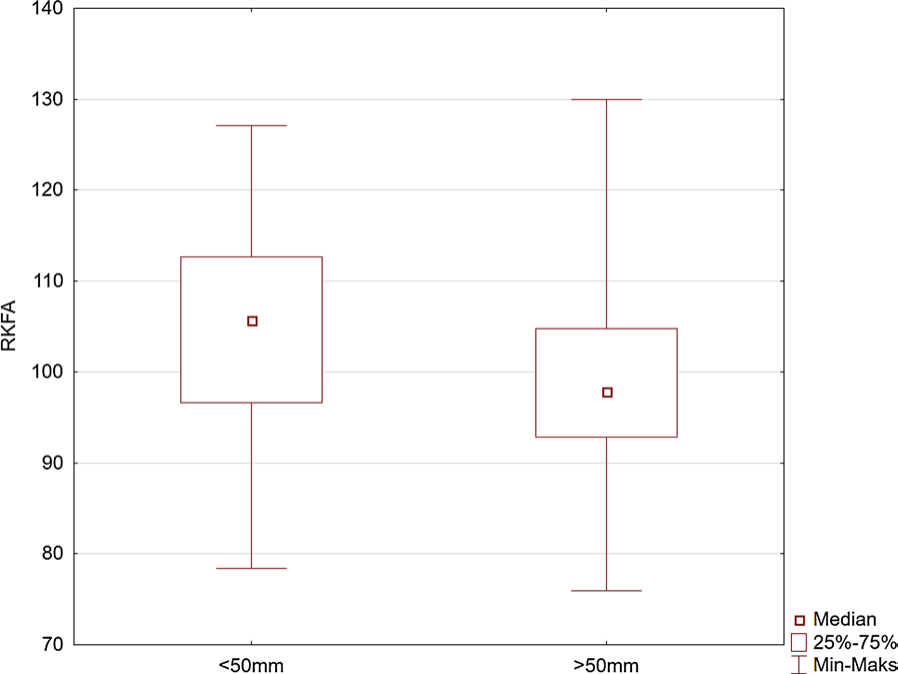
Comparison of the values of the right knee flexion angle (RKFA) for the maneuver with deep (> 50 mm) and too shallow (< 50 mm) chest compression

**Fig. 7:**
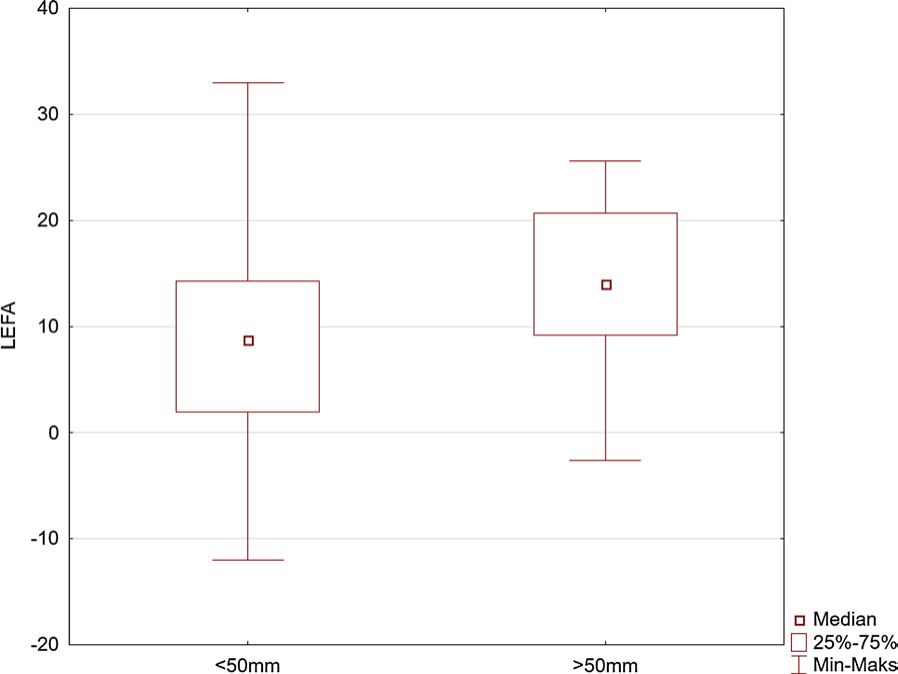
Comparison of the values of the left elbow flexion angle (LEFA) for the maneuver with deep (> 50 mm) and too shallow (< 50 mm) chest compression

**Fig. 8:**
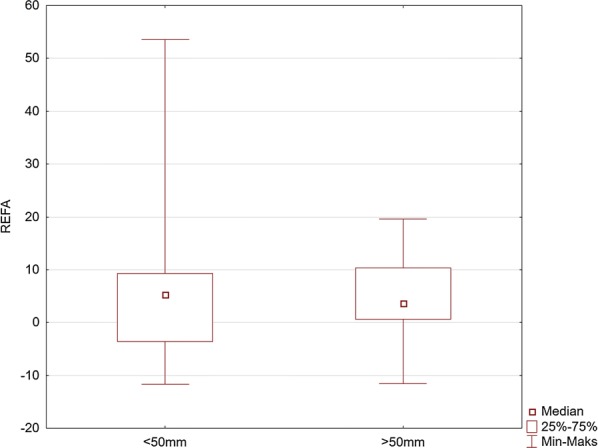
Comparison of the values of the right elbow flexion angle (REFA) for the maneuver with deep (> 50 mm) and too shallow (< 50 mm) chest compression

**Fig. 9:**
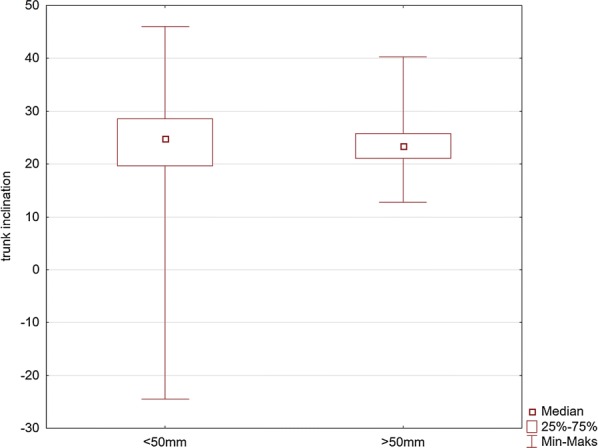
Comparison of the values of the angle of the trunk inclination for the maneuver with deep (> 50 mm) and too shallow (< 50 mm) chest compression

The analyses of the flexion angles of the trunk, knees, and elbows are shown in Figs. 5, 6, 7, 8, and 9.

## Discussion

The proper CC depth and rate are significant elements of CPR [25]. Achieving the required frequency of compressions does not seem to be problematic. In many papers, there are lists of pieces of music with the tempo corresponding to the recommended frequency of CC. The appropriate rate of compressions ensures moving to the rhythm of music. Unfortunately, there are no precise data on how to ensure the adequate chest deflection. The necessity of CPR, often lasting several or more minutes with adequate depth and frequency of compressions, undoubtedly requires from the rescuer to have appropriate characteristics of physical fitness such as strength and stamina. Chest compression is normally performed in the kneeling posture with hands on the chest of the rescued person with simultaneous maintenance of the upper limbs in the straight position perpendicular to the ground. From a biomechanical point of view, the body of the rescuer in this position forms a biokinematic chain, supported on hands and knees, which consists of nine segments (hands, forearms, arms, torso, and upper legs). The key question is how to position individual segments to provide the required CC with deflection of 5 cm. The depth of CC is influenced by the rescuer’s body mass, their experience, and the above-mentioned physical fitness and physical condition related to fatigue increasing over time [26–28]. Quick rotations of rescuers performing CC that are recommended every 1–2 min may reduce fatigue and improve the quality of CPR [28–30]. Nevertheless, we observed difficulties in achieving and maintaining the correct depth and rate of CC also in the experienced rescuers. That is why, in our study, we adopted the minimum CC of the manikin at > 40 mm [31]. To identify predictors that enhance the quality of CC, the kinematic parameters of the rescuers were analyzed during resuscitation. The multiple regression model demonstrated that significant elements of the rescuer’s kinematics facilitating the achievement and maintenance of the correct depth were the following: LEFA, LKFA, and PL. The time of the maneuver was also significant. However, it was not further analyzed as it is not an element of kinematics. Three significant elements of the kinematics of the rescuer’s movement during CPR were assessed in terms of their possible use as predictors influencing CC. The PL, which is an indirect parameter, allows the assessment of the range of the movement of the shoulder girdle (expressed by the movement of spinal segment C7-Th1) in the planes similar to horizontal and sagittal planes of the chest of the manikin. However, it does not allow the assessment of CC force. Segments indicated by PL were the result of the rescuer’s movement over the chest—forward, backward, and lateral movements over the support point on the chest, which did not allow the detailed determination of the angle with which the vector of compression force moved. In our opinion, the movement indicated only the dynamics of the rescuer’s movement over the area of CC during the maneuver. The PL was longer when CC was deeper.

The LEFA increased significantly with deep CC. However, we are of the opinion that arm flexion is a factor which absorbs the compression force and does not allow to define CC force. This parameter, as in the case of PL, should be treated as the indicator of the dynamics of the performed maneuver. Our previous study and the suggestions of other authors demonstrated that the rescuer’s body mass is a significant factor allowing deeper CC [26, 32, 33]. Therefore, in our opinion, the analysis of the activity of upper limbs demonstrated that it was impossible to maintain completely straight arms during CC, which is contrary to the other studies. When the CC force increased at the time of the movement of the rescuer’s trunk above the point of support, the LEFA increased due to the fact that the rescuer was unable to keep arms straight or to have stretched muscles of the shoulder girdle. In addition, in another study assessing the kinematics of the rescuer, no significant differences were found regarding the extent of arm movement during resuscitation. However, in that study, another method for the assessment of the kinematics of the rescuer was used [34, 35]. We believe that it can be also confirmed by the observed difference in the depth of CC, depending on the knee flexion angle. Smaller angles of the flexion of both knees by 7–8° at the time of deeper CC allowed to move the rescuer’s trunk similar to the sagittal axis running through the point of the support—placing of the dominant hand on the chest and the C7-Th1 segment, which influenced the force of CC. If we assume that, according to the force vector distribution, the value of the vertical force *F*cos (*α*) is decreased by approximately 10% with a deviation up to 25°, and then, the force increases to 98% of its maximum value when the decrease in the deviation angle is reduced to approximately 10°. Both PL and EA indicated by the sensor placed over the C7-Th1 spinal segment indicate the deviation of the axis of the compression force vector at the time of the movement over the support point on the chest. Considering the above, we believe that the indicated longer PL segments account for achieving the expected depth of CC as the reflection of the deflection of the support point under the influence of the body mass of the rescuer on the chest with the arms straight. With the same length of the limbs and raising the hips of the rescuer, change in the angle of the knee flexion is the only measurable predictor accounting for the influence of the rescuer’s position on the depth of CC. On the basis of the presented results, we believe that effective CC and thereby the quality of CPR is possible due to the movement of CC force vector over the support point. During the dynamic movement of the body mass of the rescuer over the sternum, it is possible to completely use CC force. The failure to achieve the recommended CC depth of 50 mm by the majority of the participants was probably related to the setting of a relatively high resistance of the manikin’s compression at 9.0 N/cm^2^ or errors in the technique of some participants.

Our study indicates the importance of knee and elbow flexion angles. For both groups of rescuers (reaching deflection > 50 mm and < 50 mm), the angle of the trunk inclination to the horizontal plane was 23.4°. The values of knee and elbow flexion angles in the group of rescuers who correctly performed CC (deflection > 50 mm) were statistically significantly different compared to the group of rescuers who achieved < 50 mm chest deflection. Our study showed that the knee angle should be higher than the right angle. At the same time, the upper limbs should be slightly bent at the elbow joints. The mean values of the knee flexion angle in the group of rescuers performing correct CC were 97° for the right limb and 109° for the left limb. The elbow flexion angles were 14.1° for the left limb and 3.7° for the right limb. Therefore, based on the obtained results, it can be assumed that the values of these angles are the reference values, which guarantee the required chest deflection. Maintaining the knee flexion angle > 90° during CPR ensures the center of the upper body weight to be placed forward, which guarantees stronger pressure force on the chest. In turn, slight elbow flexion at the level of several degrees requires less muscle activity of the upper limb than in the case of full elbow strengthening, thus guaranteeing lower fatigue during the procedure. In addition, slight elbow flexion provides a gentle increase in pressure force on the chest and absorbs overloading of the rescuer’s locomotor system.

Analyzing the results of the obtained examinations, it is also worth noting that the position of the rescuer’s body is asymmetrical during resuscitation. There are statistically significant differences in the positioning of the right and left sides of the body, i.e., of the upper and lower limbs. The asymmetrical position of the rescuer results in uneven loading of the right and left parts of the body, which, in the case of rescuers who often perform CC, may cause negative degenerative changes in the locomotor organ.

It is difficult to verify our observations due to the fact that only one report analyzed the kinematics of the rescuer and compared the depth of CC, depending on the change of kinematic parameters in three positions, i.e., in kneeling and standing positions next to the manikin placed at a different height above the ground [34, 35]. The results of that study did not correspond to our results and the lack of similar reports does not allow further detailed discussion. Therefore, the study has unique features and the presented results can be used as referential for the other similar studies related to this issue.

## Conclusions

The obtained results of the kinematics of the rescuers during cardiopulmonary resuscitation indicate what the correct positioning of the body of the rescuer should be like during the maneuver to ensure adequate effectiveness of the procedure. Obviously, the practical implementation of the results may increase the effectiveness of CC performed by experienced rescuers through the correction of motor habits, whereas, in the group of young rescuers, it should ensure the acquisition of normal locomotor patterns during resuscitation.

Raising the rescuer’s hips allowed the movement of the rescuer’s body mass over the point of sternal compression by increasing the value of the CC force vector, thus allowing deeper CC. Thus, permanent maintenance of knee flexion > 90° during CC allows the rescuer’s center of the body weight to be placed over the pressure point of the sternum, guaranteeing stronger pressure force on the chest.

During effective CC, the rescuer was not able to maintain arms straight and, in consequence, elbow flexion was observed. It, however, did not influence the quality of the maneuver. However, the deflection of the arms in the elbow joints by several degrees during CC results in a gentle increase in the pressure force on the chest and absorbs overloading of the rescuer’s locomotor system.
